# The effect of intravenous lidocaine on postoperative cognitive dysfunction: a systematic review and meta-analysis

**DOI:** 10.1186/s12871-023-02202-0

**Published:** 2023-09-05

**Authors:** Chuan Geng, Baoji Hu, Jihong Jiang, Yunhe Zhang, Weiqing Tang, Mengzhi Pan, Leilei Sun, Peifen Chen, Hengyue Wang

**Affiliations:** 1 Department of Anesthesiology, Fengxian People’s Hospital, Fengxian County, Xuzhou City, 221700 Jiangsu Province China; 2 Department of Anesthesiology, Shanghai Pudong Hospital, Fudan University Pudong Medical Center, Shanghai, 201399 China; 3 Department of Anesthesiology, Shanghai General Hospital, Shanghai Jiao Tong University School of Medicine, Shanghai, 200080 China; 4 Department of Centre ICU, Shanghai East Hospital, School of medicine, Tongji University, Shanghai, 200085 China; 5 Department of Respiratory Diseases, The Third People’s Hospital of Shenzhen, the Second Affiliated Hospital of Southern University of Science and Technology, Shenzhen, 518112 Guangdong China; 6 Faculty of Anesthesiology, Changhai Hospital, Naval Medical University, Shanghai, 200433 China

**Keywords:** Lidocaine, Postoperative cognitive dysfunction, Meta-analysis, Intravenous, Elderly

## Abstract

**Background:**

Postoperative cognitive dysfunction (POCD) has been reported as a significant complication in elderly patients. Various methods have been proposed for reducing the incidence and severity of POCD. Intravenous lidocaine administration has been reported in the literature to reduce POCD, but the effect of lidocaine remains controversial.

**Methods:**

We screened Medline, Embase, Cochrane Library, and China National Knowledge Infrastructure (up to April 2022) databases following a search strategy for intravenous lidocaine on POCD. We also screened related bibliographies on lidocaine for POCD. Ten articles comprising 1517 patients were selected and analyzed. We divided the postoperative follow-up period as follows: short term (<30 days), medium term (30–90 days), and long term (>90 days).

**Outcomes:**

We found that lidocaine could attenuate the overall incidence of POCD, especially in the short term. There were no differences between lidocaine and placebo on the overall severity of POCD.

**Conclusion:**

Lidocaine administered intravenously could attenuate the overall incidence of POCD and its severity in the short term.

**Supplementary Information:**

The online version contains supplementary material available at 10.1186/s12871-023-02202-0.

## Introduction

Postoperative cognitive dysfunction (POCD), which reflects a negative change in an individual’s cognitive trajectory, has increasingly been recognized as a complication in elderly patients. Cognitive decline lasts for months to years. The incidence of POCD varies from 1.43 to 59% in surgical patients [1–3]. Too many factors contribute to the wide-range incidence, including higher rates of cerebrovascular and myocardial injury, infection and respiratory complications, and diverse surgical populations [4]. In addition, POCD is currently a hypothetical phenomenon for which there is no International Statistical Classification of Disease (ICD-9) code and no Diagnostic and Statistical Manual of Mental Disorders (DSM-IV) code, and nonstandard testing may also amplify the range [5, 6]. The potential pathogenesis of POCD involves neuroinflammation and oxidative stress secondary to anesthesia and surgery [7, 8]. Post-surgery inflammation is characterized by increased levels of inflammatory cytokines and mediators and vascular permeability [9]. Excessive inflammation can disrupt the body’s immune system, potentially leading to certain inflammation-related conditions [10].

Lidocaine, a commonly used local anesthetic, crosses the blood-brain barrier (BBB) and exerts anti-inflammatory effects by inhibiting the expression of pro-inflammatory cytokines and the release of histamine [11–13]. Systemic administration of lidocaine has been reported to decrease the occurrence of cognitive dysfunction in the postoperative period [14]. However, Mathew et al. argued that subjects receiving lidocaine were more likely to experience cognitive decline, possibly because of altered lidocaine metabolism [15]. To address the controversies and scant evidence regarding the neuroprotective effect of lidocaine, we conducted the present meta-analysis and systemic review to determine whether the administration of lidocaine could reduce cognitive dysfunction in patients.

## Methods

### Literature retrieval and research selection

We followed the guidelines of the Preferred Reporting Items for Systematic Reviews and Meta-Analysis (PRISMA) [16] and assessing the methodological quality of systematic reviews (AMSTAR) to report our results. Two investigators independently searched the Medline, China National Knowledge Infrastructure, Embase, and Cochrane Library (up to April 2022) databases for randomized control trials that reported associations between lidocaine and postoperative cognitive dysfunction. The Boolean operator between keyword groups was “AND” and “OR” within the groups. Search terms were created by combing the following medical subject headings (MeSH terms): (“Cognitive Therapy” OR “Cognition Disorders” OR “Cognition” OR “Neuropsychology” OR “Neuropsychological Tests” OR “Cognitive Impairment” OR “delirium” OR “postoperative cognitive dysfunction”) AND (“Lidocaine”). The search strategy on Medline is listed in the Appendix, and adjusted slightly in the different databases. To ensure a comprehensive literature search, no languages were restricted, and we also reviewed the bibliography of relevant publications. When the required data were unclear or missing, the author(s) was contacted.

### Inclusion and exclusion criteria

The inclusion criteria were as follows: patients were under operation, lidocaine was administered systematically as an intervention, the postoperative cognitive dysfunction was compared before and after operation, the endpoint of the study was postoperative cognitive dysfunction, and studies were randomized control studies. The exclusion criteria were as follows: case reports, comments, reviews, or other types of literature; age less than 18 years; lidocaine was administrated locally or intramuscularly; original data could not be obtained; animal studies; and low-quality studies (Jadad score < 3).

### Data extraction and quality assessment

Two authors independently screened the titles and abstracts of the studies and reviewed their full texts of selected studies using structured extraction forms. The characteristics of the included studies were as follows: initial of the first author, publication year, language, geographical location, placebo, participants (sex, age, sample size, history of cognitive dysfunction or psychotropic medication), intravenous lidocaine regimen, cognitive measurement, and follow-up time of assessment. Disagreements were resolved by a third rater, who was approved by a board-certified anesthetist not involved in the initial data extraction.

The occurrence of POCD was defined as at least a 1SD decline in the postoperative score compared with the preoperative score in the included studies. Continuous cognition variables measured using the Mini Mental State Examination (MMSE), information-memory-concentration test (IMCT), and neuropsychological (NP) tests were all included in the meta-analysis. Based on the follow-up assessment, we defined it as short term (< 1 month), medium term (1–3 months), and long term (> 3 months).

The modified Jadad scale [17] was used to evaluate article quality. Although some have argued that the Jadad score is a simplistic measure that does not characterize all elements of trial quality, it is still perhaps the most common measure of trial quality, and it offers the prospect of objectivity, which is much more efficient than some other subjective methods. The modified Jadad scale comprises a five-point scale. The scale was defined as follows: (i) was the study described as randomized? “yes or no”; award a bonus point if the method of randomization is appropriate (e.g., computer-generated, score 2), deduct one point if the method of randomization is inappropriate (score 1)–no randomization score was 0; (ii) was the study described as double-blind? “yes or no”; award a bonus point if the method of double blinding is appropriate (e.g., identical placebo, score 2), deduct one point if the method of double blinding is inappropriate (score 1)–no double blinding score was 0; (iii) Was there a description of withdrawals and dropouts? “yes (score 1) or no (score 0).” The scale scores can range from 0 to 5 points, with higher scores indicating better quality. Studies with a score of ≥ 3 were considered high-quality trials, and those with scores of < 3 were considered low-quality trials [18].

### Statistical analysis

The analyses were conducted on an experiment-to-control basis. A fixed-effects model was used, and a random-effects model was employed in the case of significant heterogeneity (P-value of chi-square test less than 0.10 and I^2^ greater than 50%). This means that variables with a P-value of chi-square test less than 0.10 were considered heterogeneous, the amount of total variance was more than we would expect based on within-study error, and a random effect model was assumed. To provide a more conservative estimation, random rather than fixed effect models were adopted because the former can explain heterogeneity between studies. When the heterogeneity was high, subgroup and sensitivity analyses were conducted to explore the sources of heterogeneity. Potential sources of heterogeneity were identified using sensitivity analyses conducted by omitting one study in each turn and investigating the influence of a single study on the overall pooled estimate. The “risk of bias” according to the Cochrane Handbook was used for quality evaluation of the included literature, including adequate sequence generation, allocation concealment, blinding, incomplete outcome data addressed, free of selective reporting, and other biases. The evaluation grade included three levels of “yes, unclear, and no,” and finally, a risk assessment chart of bias was formed. Publication bias was evaluated by using funnel plots. Egger’s test was used to evaluate potential publication bias in the case of a few trials included in the meta-analysis. Statistical significance was set at p < 0.05. Data are presented as the mean ± standard deviation for continuous variables and as proportions (%) for categorical variables. Dichotomous results were analyzed using the Mantel-Haenszel (MH) method. Risk ratio (RR) and 95% confidence interval (CI) were calculated. The mean difference was calculated for continuous results. All statistical analyses were performed using the Statistical Program for Social Sciences 26.0 (SPSS, Inc., Chicago, IL, USA), and meta-analysis was performed using Review Manager 5 (RevMan, The Cochrane Collaboration, Oxford, United Kingdom). We used the GRADE profiler (GRADEpro, McMaster University and Evidence Prime Inc. Hamilton, Ontario, Canada) to evaluate the quality of the RCT evidence, including the overall risk of bias, inconsistency, indirectness, imprecision, and publication bias. Egger’s test was performed using StataMP 17 (Stata Corporation LLC, College Station, TX, USA). Any inconsistencies in the assessment results were resolved through negotiation following the inclusion and exclusion criteria. For a given study, assessed POCD at different follow-up time points, we may divide the study into multiple studies. Because we have to divide the study into subgroups, based on the follow-up time point. Cohen’s kappa statistic was used to measure the level of agreement between two researchers who classified items into mutually exclusive categories. The formula for Cohen’s kappa was calculated as kappa = (Po-Pe)/(1-Pe), where Po is the relative observed agreement among raters and Pe is the hypothetical probability of chance agreement.

## Results

### Study selection

**Fig. 1:**
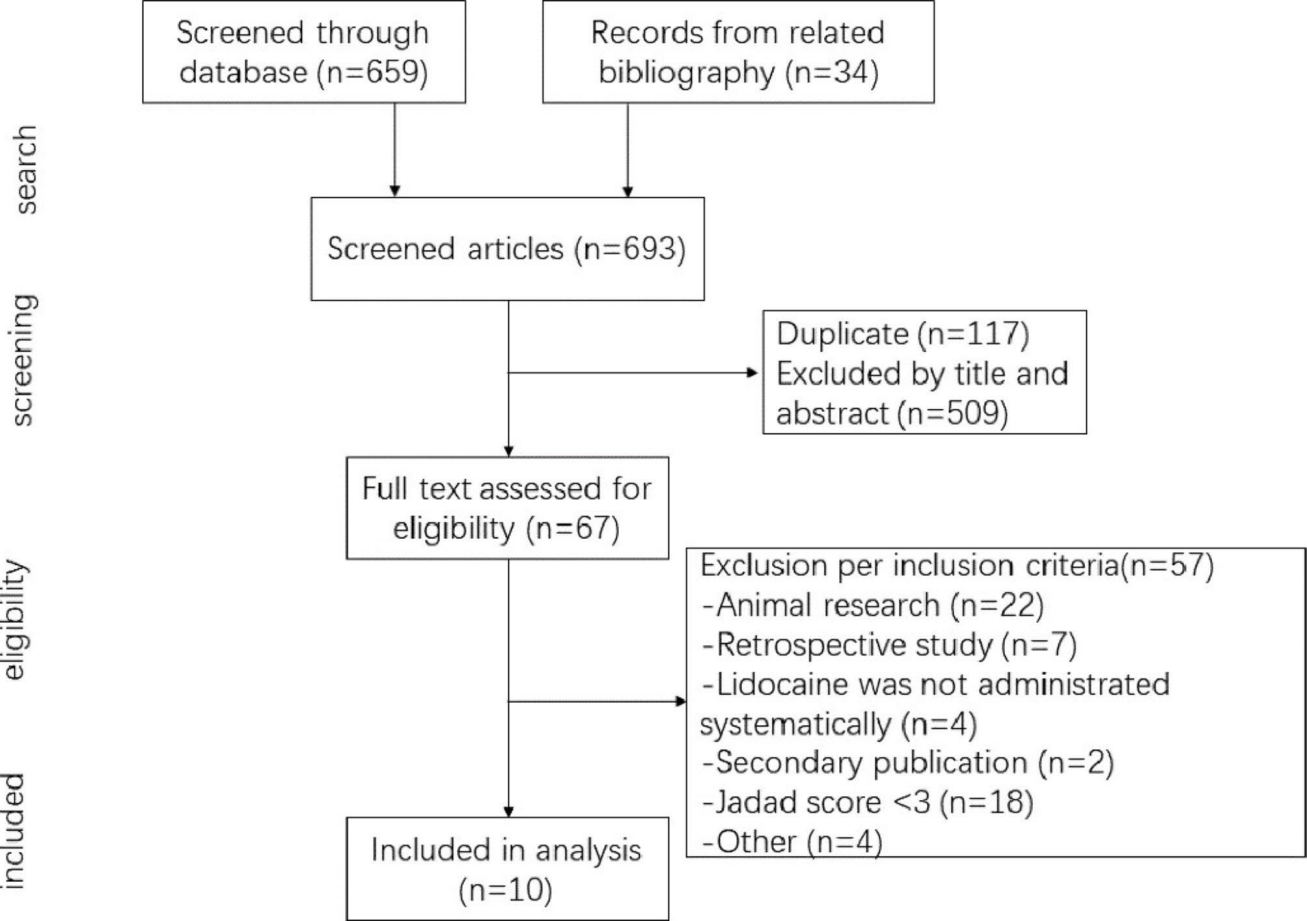
PRISMA diagram showing selection of articles for review of POCD.

Cohen’s kappa value was 0.997 in this study. Following the search strategy, the study yielded 659 publications and 34 publications from the bibliography of related articles. Sixty-seven full articles were assessed for eligibility after the removing of 117 duplications, and 509 were discarded for the title and abstract. Fifty-seven articles were excluded for animal research, retrospective study, lidocaine used locally, secondary publication, low-quality publication, and other reasons. Ten RCTs [11, 14, 15, 19–25] including 1517 patients, met the inclusion criteria. The details of the PRISMA search strategy are shown in Fig. 1.

Of the 10 included studies, two [20, 25] were published in Chinese with English abstracts and all others in English. Regarding geographical distribution, there were six trials [14, 20, 22–25] from Asia, two [11, 15] from North America, and two [19, 21] from Oceania. Regarding publication quality, four trials [11, 15, 22, 24] had a full score on the Jadad scale, four trials [14, 21, 23, 25] had four points, and two trials [14, 19] had three points.

### Characteristics of included studies

A total of 10 RCTs were included in the analysis. The baseline characteristic of included studies were summaried in Table 1. Of all the patients, 754 received lidocaine intravenously, and 763 participants served as controls. In one trial, patients in the comparator arm received placebo treatment with 5% dextrose, and in other trials received 0.9% normal saline. Lidocaine was used as a bolus around induction of anesthesia or at the opening of the pericardium, followed by continuous pump injection in nine trials [11, 14, 15, 19–21, 23–25]; one trial [22] used a single injection. Bolus doses of lidocaine (0.5, 1.0 or 1.5 mg/kg) followed with different infusion regimens. The serum lidocaine levels varied strongly among the studies.

**Table 1:** Baseline characteristic of included studies

	Mitchell et al. 1999		Wang et al. 2002		Mathew et al. 2009		Mitchell et al. 2009		Peng et al. 2016		Chen et al. 2015		Klinger et al. 2019		Hashemi et al. 2013		Zhu et al. 2015		Wang et al. 2004	
	Lid	Con	Lid	Co	Lid	Co	Lid	Co	Lid	Con	Lid	Con	Lid	Con	Lid	Con	Lid	Con	Lid	Con
Age*	56.9 ± 8.9	54.4 ± 9.7	57.8 ± 9.7	59.3 ± 9.4	61.7 ± 11.9	61.4 ± 13.9	61.5 ± 9.6	58.1 ± 11.4	45 ± 9	44 ± 10	71.3 ± 2.0	71.8 ± 1.9	67 ± 9.1	67 ± 9.5	66 ± 1	67 ± 2	71.9 ± 3.8	73.1 ± 4.2	58 ± 10	59 ± 9
Male (%)	17 (60.7)	14 (51.9)	42 (97.7)	44 (97.8)	83 (72.8)	85 (67)	60 (74.1)	63 (81.8)	20 (50)	19 (47.5)	25 (62.5)	13 (57.7)	151 (71.6)	160 (76.6)	27 (77.1)	28 (80)	13 (43.3)	13 (43.3)	42 (97.7)	44 (97.8)
Sample size	28	27	43	45	133	144	81	77	40	40	40	40	211	209	35	35	30	30	43	45
Education*	9.78 ± 3.0	9.81 ± 2.4	9.6 ± 3.5	10.1 ± 3.9	13.3 ± 3.4	12.7 ± 3.3	NA	NA	12.25 ± 3.43	12.75 ± 3.19	NA	NA	14.3 ± 2.99	15.3 ± 2.99	NA	NA	5.9 ± 2.3	6.2 ± 2.4	10 ± 3	10 ± 4
MMSE score	NP test	NP test	NP test, occurrence	NP test, occurrence	Cognitive index, Occurrence	Cognitive index, Occurrence	Occurrence	Occurrence	28.65 ± 0.77	29 ± 1.54	28.77 ± 1.85	28.19 ± 0.98	Cognitive index, Occurrence	Cognitive index, Occurrence	25.9 ± 4	25.7 ± 3.9	27.3 ± 1.7, NP test, occurrence	26.9 ± 1.4, NP test, occurrence	NP test, occurrence	NP test, occurrence
surgery	CABG with CPB, valve surgery		CABG with CPB		CABG with CPB		CABG with CPB, valve surgery		Supratentorial craniotomy for tumor surgery		Spine surgery		CABG, CABG with valve, valve		Urologic and orthopedic		Gastrointestinal tumor surgery		CABG with CPB	
Follow-up	10d, 10wk, 6mo		9d		6wk, 1yr				24 h, 1wk, 1mo, 3mo, 6mo		3d		6mo, 1yr		leaving PACU, 6 h, 24 h		2d		9d	
Jadad score	3		4		5		4		5		4		5		5		4		3	

*Data are reported as mean ± SD;Lid indicates Lidocaine; Con indicates Control

Studies were conducted in patients undergoing either cardiac surgery [11, 14, 15, 19–21] including CABG with or without CPB; CABG with valve, valve, or supratentorial craniotomy surgery [24]; urologic and orthopedic surgery [22]; spine surgery [23]; or gastrointestinal tumor surgery [25]. The data and conclusion from eligible studies were summarized in Table 2.

**Table 2:** Summary of data and conclusions from eligible studies

Studies	Design	placebo	Trial medication	Plasma concentration	Test battery	Drop out	conclusion
Mitchell et al. 1999	RCT	5% dextrose	Began at the induction, 1 mg/kg bolus over 5 min, 240 mg for the first hour, 120 mg for the second hour, 60 mg/h for 46 h	6–12 (µmol/L)	6 tests with 11 subscales	9 (14.06%) patients lost after randomization, 4 (12.5%) in lidocaine and 5 (15.63) in placebo	No difference
Wang et al. 2002	RCT	Saline	1.5 mg/kg bolus over 5 min at the opening of pericardium, 4 mg/min till the end of surgery; 4 mg/kg to the priming solution of CPB	5.52 ± 1.18 (µg/ml)	7 tests with 9 subscales	30 patients (25.42%) lost, 16 (26.23%) in placebo, and 14 (24.56%) in lidocaine	Decline
Mathew et al. 2009	RCT	Saline	1 mg/kg bolus, 4 mg/min for first hour, 2 mg/min for second hour, 1 mg/min for 46 h	2.45 ± 0.93(mg/mL)	5 tests	105 (37.91%) patients lost after randomization, 50 (34.72%) in placebo and 55 (41.35%) in lidocaine	No difference
Mitchell et al. 2009	RCT	Saline	1 mg/kg bolus over 5 min at induction, 2 mg/min for 2 h, 1 mg/min for 12 h.	6–12 (µmol/L)	7 tests and self-rating	51 (32.28%) patients lost, 24 (31.17%) in placebo and 27 (33.33%) in lidocaine	No difference
Peng et al. 2016	RCT	Saline	1.5 mg/kg bolus after induction, 2 mg/kg/h till the end of surgery	NA	MMSE, IMCT	14 patients (14.89%) lost, 6 (13.04%) in lidocaine and 8 (16.67%) in placebo	No difference
Chen et al. 2015	RCT	Saline	1 mg/kg bolus over 5 min after induction, 1.5 mg/h till the end of surgery	NA	MMSE	No patients lost after randomization	Improve cognition
Klinger et al. 2019	RCT	Saline	1 mg/kg bolus after induction, 48 µg/kg/min for the first hour, 24 µg/kg/min for the second hour, 10 µg/kg/min for 46 h	Less than 5 µg/ml	5 tests	101 (21.13%) patients lost, 45 (18.99%) in placebo and 56 (23.24%) in lidocaine	No difference
Hashemi et al. 2013	RCT	Saline	1.5 mg/kg before extubation	NA	MMSE	No patients lost after randomization	No difference
Zhu et al. 2015	RCT	Saline	0.5 mg/kg bolus after induction, 0.5 mg/kg/h till to the end of surgery	NA	5 tests	No patients lost after randomization	Decline
Wang et al. 2004	RCT	Saline	1.5 mg/kg bolus at opening the pericardium, 4 mg/min till the end of surgery; 4 mg/kg to the priming solution of CPB	5.54 ± 1.23 (µg/ml)	9 tests	30 (25.42%) patients lost, 16 (26.23%) in placebo and 14 (24.56%) in lidocaine	Decline

MMSE indicates Mini-Mental State Examination; IMCT, information-memory-concentration test; NA, not available

The overall age of patients was 62.72 ± 11.56 years, and there were no differences between lidocaine and placebo (62.89 ± 11.02 vs. 62.54 ± 12.08, p = 0.57). The proportion of male patients receiving lidocaine (70.18%) and placebo (69.80%) was not significantly different. The overall education was 12.6 ± 4.0 years from the available studies, and there were also no differences between the lidocaine (12.44 ± 3.9) and placebo (12.76 ± 4.1) groups. The authors addressed the dropout situation in eligible studies, and 70% (7 out of 10) of the studies reported lost patient numbers. We found that 26.0% (340 of 1307) of patients were lost after randomization. There were no differences between the lidocaine and placebo groups (27.5% vs. 24.5%, p = 0.19). Because of the different infusion strategies of lidocaine, the serum lidocaine level was difficult to compare between the studies. It can be considered safe and effective with respect to plasma concentration [26, 27].

Of the included studies, six studies [14, 19, 20, 22, 24, 25] assessed the occurrence of POCD, and four studies [22–25] elevated the continuous score via MMSE (or IMCT, HRSD, HAMA), including one trial [25] for baseline only, and another four trials [14, 19, 20, 25] via NP test with different scales.

### Incidence of POCD

The overall incidence of POCD was 33.31%, of which 30.12% and 36.40% were for lidocaine and placebo, respectively. Heterogeneity was calculated (Chi^2^ = 36.35, I^2^ = 6%). The incidence of POCD in lidocaine was significantly lower than that in the placebo group, with MH RR as 0.84 (95% CI: 0.76 to 0.92).

**Fig. 2:**
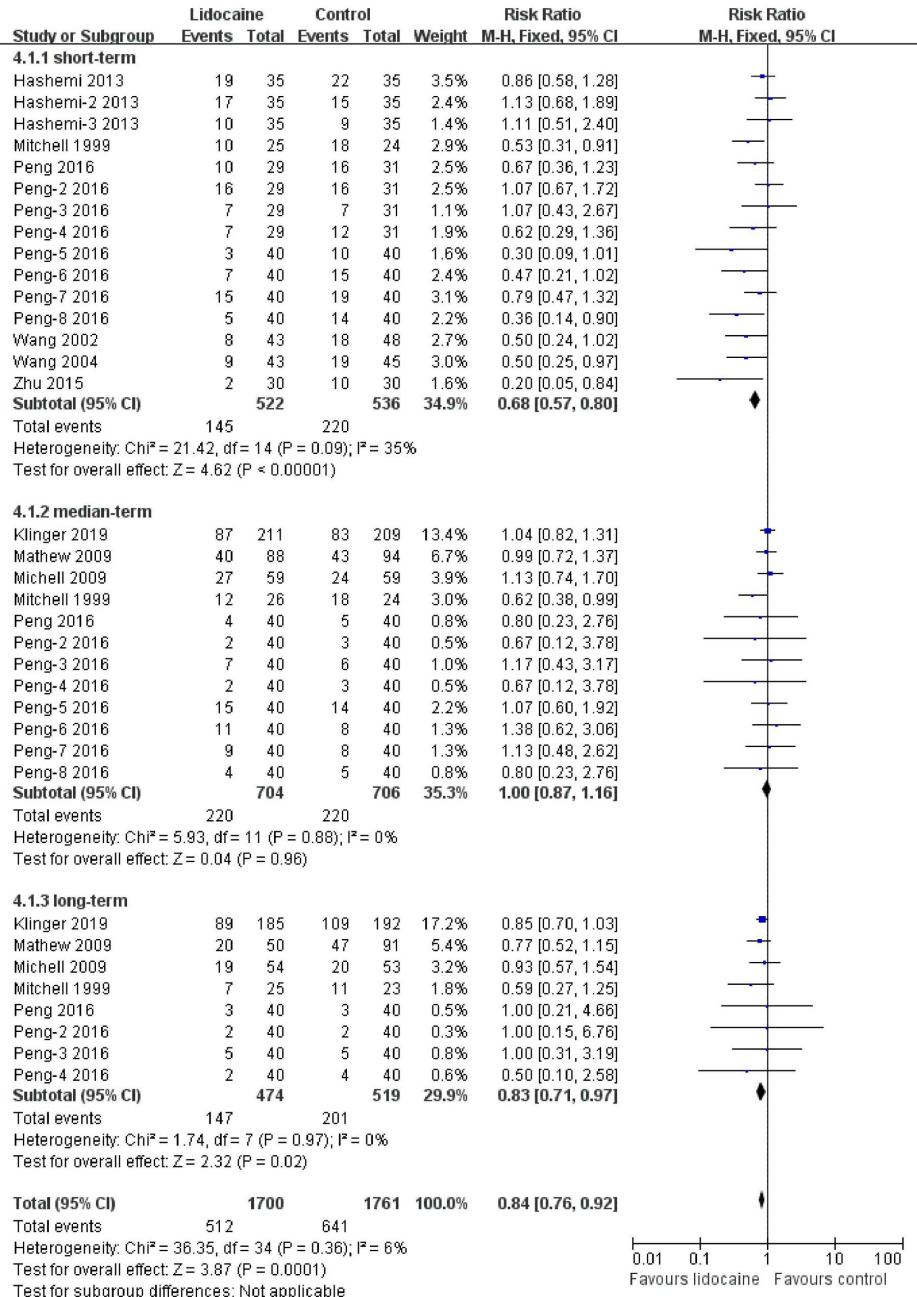
Forest plot of the incidence of POCD.

Studies have reported an elevated occurrence at various time points. We defined POCD into three segments (short-, medium- and long term) and analyzed its occurrence in three subgroups. Six studies assessed the incidence of POCD in the short term period; the overall incidence of POCD in the short term was 34.50%, of which 27.78% occurred in the lidocaine group, and 41.04% occurred in the placebo group. A meta-analysis of the incidence of POCD revealed a significantly lower occurrence in the lidocaine group than in the placebo group in the short term (MH RR = 0.68, 95% CI: 0.57 to 0.80). Five studies assessed the incidence of POCD in the medium term. The overall incidence of POCD was 31.21%; with an incidence of 31.25% in the lidocaine group and an incidence of 31.16% in the placebo group. The meta-analysis did not demonstrate any differences between the groups (MH RR = 1, 95% CI: 0.87 1.16). Five studies also assessed the incidence of POCD in the long term. The incidence of POCD was 35.05% in the long term, 31.01% in lidocaine, and 38.73% in placebo. The meta-analysis revealed a significant difference between the groups (MH RR = 0.83, 95% CI: 0.71 to 0.97). Figure 2 summarizes the results of the pool analyses.

**Fig. 3:**
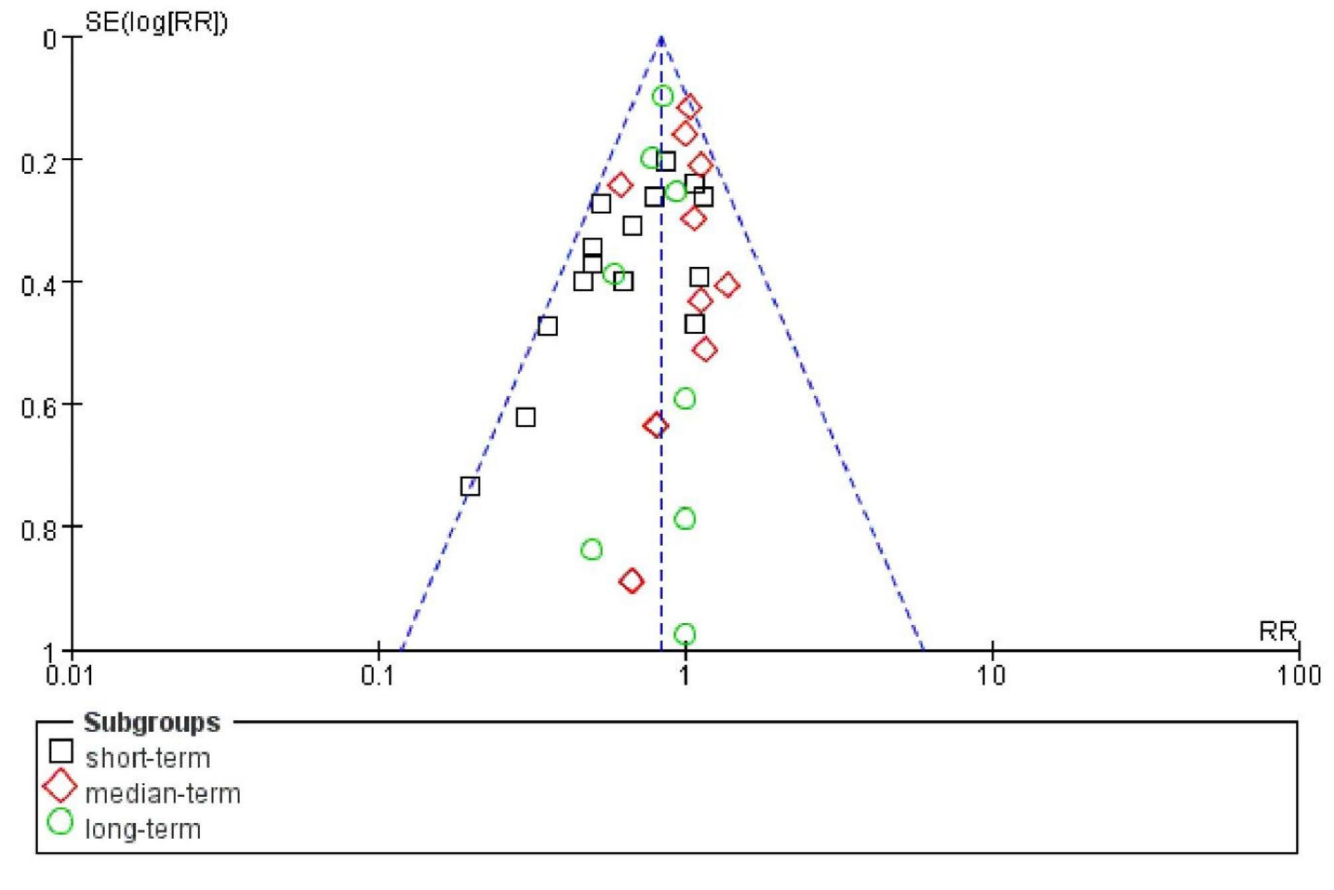
Funnel plot of the studies on the incidence of POCD.

We compared the weighted mean and standard deviation for short-, medium-, and long term meta-analysis. As a result, we did not find any differences between short term (2.39 ± 0.18), medium term (2.95 ± 1.08), and long term (3.73 ± 2.02) with p-value = 0.697. Although only six (out of ten included) studies evaluated the incidence of POCD, a battery of data (n = 35) was included in the meta-analysis. We demonstrated the publication bias via a funnel plot (Fig. 3); from the figure, we may infer that there were no obvious differences.

We performed sensitivity analysis by omitting one study. We removed the study conducted by Mathew et al. [15], as the overall loss to follow-up of the study was up to 37.91% and even to 41.35% for the lidocaine group. MH RR decreased from 0.84 to 0.83, which means that the significant association between lidocaine and POCD was not confounded by the study.

The GRADEpro assessed the overall quality of the eligible studies in the incidence of POCD and deduced a moderate-quality grade.

### The severity of POCD

**Fig. 4:**
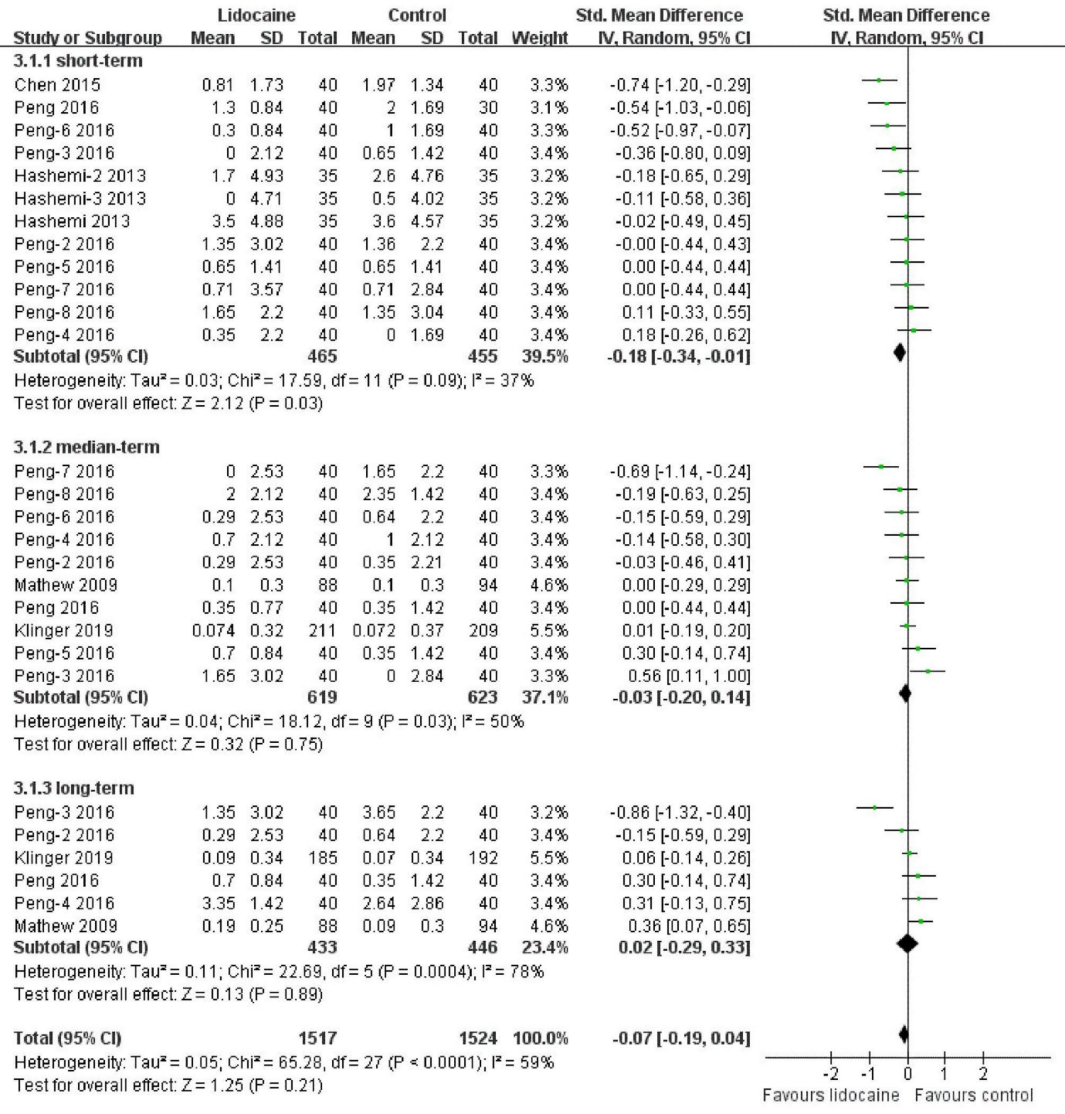
Forest plot of the severity of POCD.

**Fig. 5:**
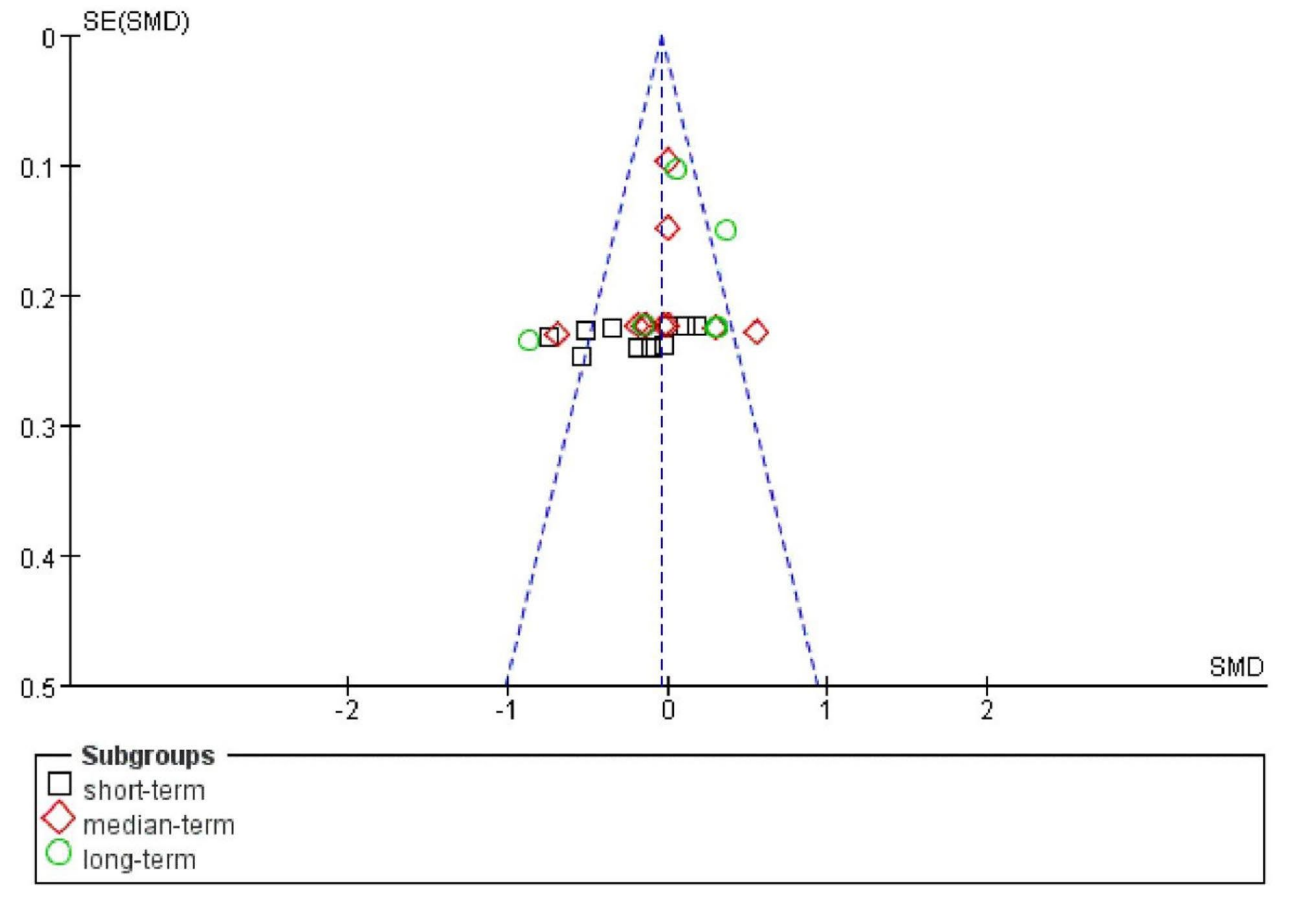
Funnel plot of the studies on the severity of POCD.

Three studies assessed the severity of POCD using continuous cognitive variables, including MMSE, IMCT, and NP tests. As the meta-analysis demonstrated, there were no differences between the lidocaine and placebo groups (p = 0.21). The overall Standardized mean difference was − 0.07 (95% CI: -0.29 to 0.04). However, in the subgroup analysis, lidocaine could attenuate the severity of POCD in the short term with a Standardized mean difference of -0.18 (95%CI: -0.34 to -0.01), but not in the medium and long term (-0.03 [95% CI: -0.2 to 0.14] and 0.02 [95% CI: -0.29 to 0.33], respectively). Figure 4 shows the pool analysis. We compared the weighted mean and standard deviation among the short term (3.31 ± 0.03), medium term (3.71 ± 0.23), and long term (3.92 ± 0.38). The results revealed no significant differences between the groups (p = 0.119). Publication bias was demonstrated using a funnel plot (Fig. 5). It can be inferred that the difference was not obvious from Fig. 5.

A sensitivity analysis was conducted by omitting one study. A battery of data from the study conducted by Hashemi et al. [22] had the highest standard error (4.93 and 4.76 for lidocaine and placebo, respectively) among RCTs. The Standardized mean difference remained at -0.07, and the scope of 95% CI changed from (-0.19 to 0.04) to (-0.19 to 0.05). The overall effect of lidocaine remained even after exclusion from the study.

The GRADEpro assessed the overall quality of the eligible studies in terms of the severity of POCD and deduced the quality grade as high.

**Fig. 6:**
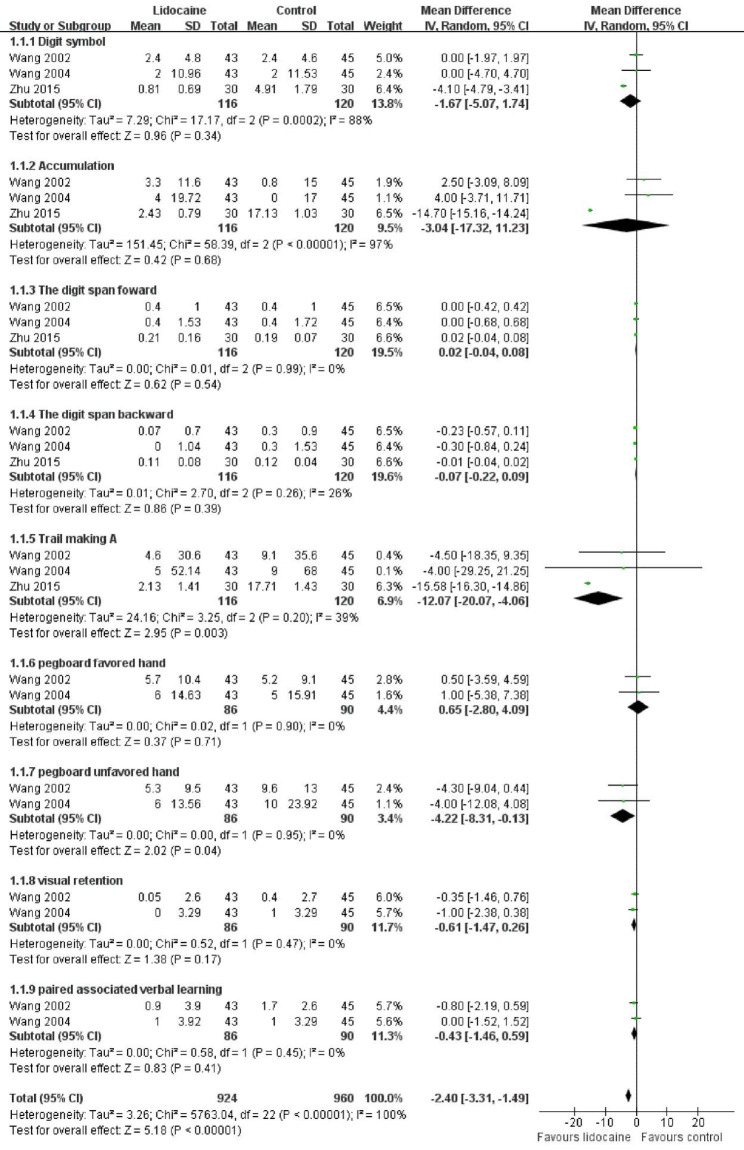
Forest plot of the severity of POCD measured via NP test

Three studies compared the influence of lidocaine versus placebo on POCD using the NP test with different scales in the short term, including digit symbol, accumulation, digit span forward, digit span backward, trail making A, pegboard favored hand, pegboard unfavored hand, visual retention, and paired associated verbal learning. We classified the scales into subgroups and performed a meta-analysis. As the results demonstrated, lidocaine could attenuate the severity of POCD in the short term with an overall Standardized mean difference of -2.4 (95%CI: -3.31 to -1.49), especially at trail making A (-12.07 [95% CI: -20.07 to -4.06]) and pegboard unfavored hand (-4.22 [-8.31 to -0.14]) (Fig. 6). Publication bias for the NP test could not be assessed by a funnel plot because there were only three studies included for meta-analysis. It is not recommended to assess publication bias by using funnel plots for fewer studies. Egger’s test was applied to analyze publication bias, and the results revealed no differences (P > 0.05).

A sensitivity analysis was conducted by omitting one study. We removed the study conducted by Wang et al. [20] because of its higher standard error in subgroups of digit symbol, accumulation, trail making A, pegboard favored hand, and pegboard unfavored hand (10.96,19.72, 52.14, 14.63, 13.56 and 11.53, 17, 68, 15.91, 23.92, in the lidocaine and placebo groups, respectively). The overall effect of lidocaine remained with the Standardized mean difference changing from − 2.4 (95% CI: -3.31 to -1.49) to -2.58 (95% CI: -3.52 to -1.64).

The GRADEpro assessed the overall quality of the eligible studies in the NP test and deduced the quality grade as moderate.

**Fig. 7:**
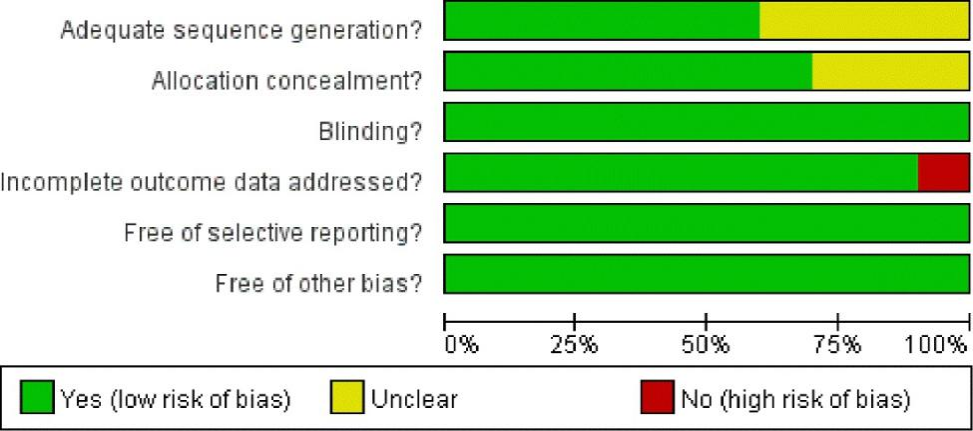
Graph of risk bias

**Fig. 8:**
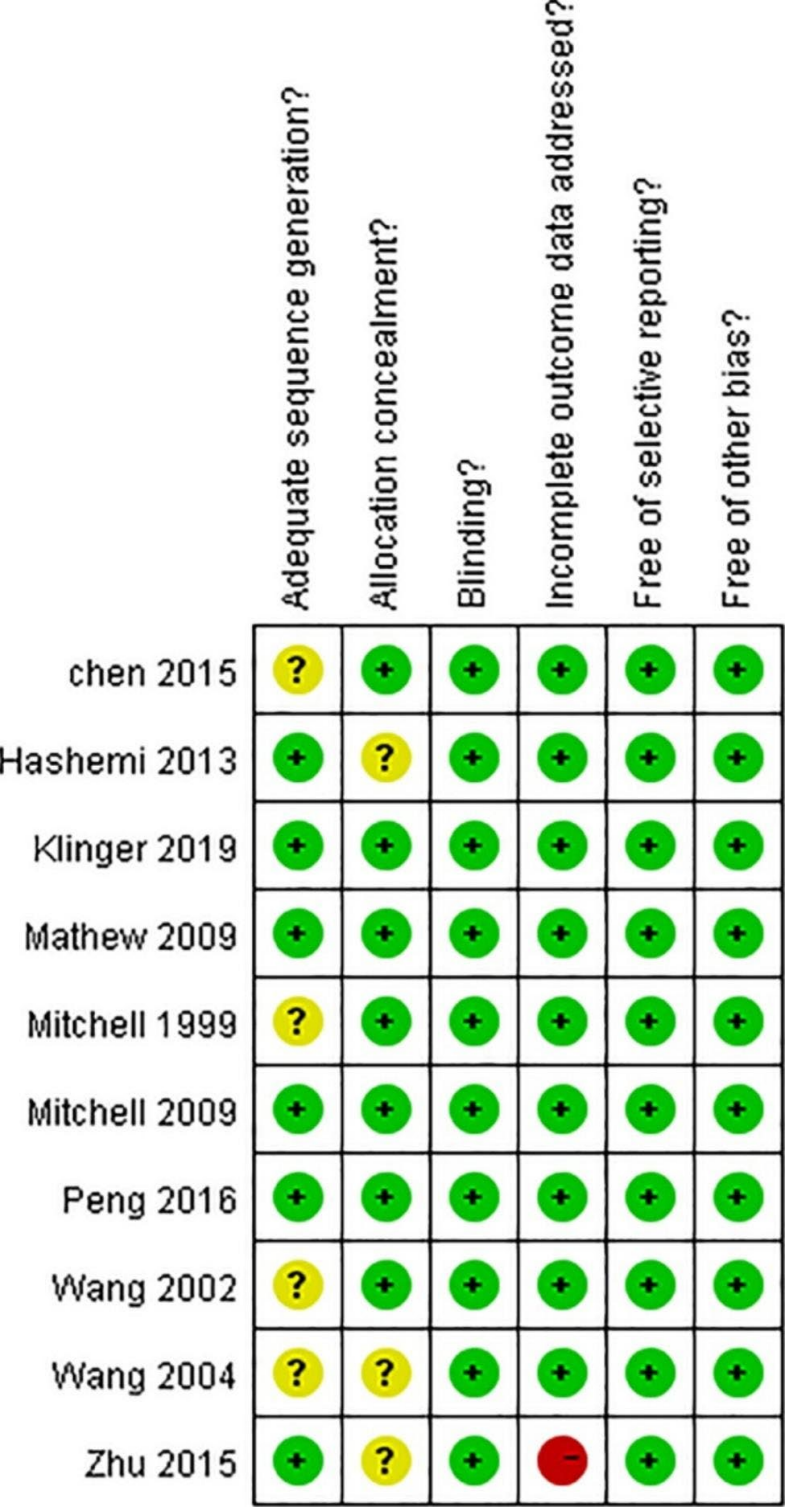
Summary of risk of bias

The risk of bias in the included studies is demonstrated in Fig. 7, and the summary risk of bias is demonstrated in Fig. 8.

## Discussion

The effectiveness of lidocaine in POCD is still unclear, and to the best of our knowledge, no previous meta-analysis studies have assessed the effect of intravenous lidocaine on the incidence and severity of POCD. We demonstrated that lidocaine treatment significantly reduced the occurrence of POCD, especially in the short and long term. A meta-analysis conducted by Baradari et al. [28] revealed that lidocaine consistently reduced the incidence of cognitive deficits significantly after cardiac surgery, particularly during the first postoperative month. Although there were no differences between lidocaine and placebo in the overall severity of POCD, lidocaine attenuated the severity of POCD in a short term subgroup meta-analysis.

Variability in the follow-up time of cognitive assessment is an important factor that complicates the interpretation of the literature. The follow-up period was covered from the discharge of the post-anesthesia care unit through one year after the surgery in the eligible studies. POCD frequently occurred in the short term [29, 30], and a previous study suggested a pattern of improvement in the short term postoperative cognitive function, which predicted a later decline [29]. We found that lidocaine decreased the incidence and attenuated the severity of POCD in the short and long term. Thus, lidocaine may be a useful agent for treating POCD.

It had been identified in the literature that risk factors for POCD include advanced age and shorter education [31]. Although the eligible studies were randomized trials, the intervention and control groups were rigorously compared under the same circumstances. We compared the age and education between the lidocaine and placebo groups and did not find any differences. This means that the biases of the two risk factors were balanced. Whether the surgical procedure is an independent risk factor for POCD remains controversial. The high rate of POCD occurrence after CABG in multiple studies [3, 29], suggests that the CABG procedure puts patients at risk of cognitive decline. However, most outcomes of CABG studies are limited by a lack of appropriate control groups. Most importantly, although cognitive changes are well documented, assessment of whether they are specifically related to the procedure itself or whether other surgical procedures would produce similar postoperative cognitive changes has been difficult. In other words, some of the short term cognitive changes after CABG may not be specific to the procedure but may also accompany other surgical procedures. POCD may be suggested as a multifactorial etiology of prolonged cardiopulmonary bypass time [32] and cerebrovascular pathology [3]. Regarding study variability, it may be said that there were no significant variables, except for lidocaine treatment. Based on the above literature, we did not set the inclusion criteria for the surgical procedure in this study.

Given that dropout can result in worse outcomes [33] and even 26% dropout after randomization, there were no differences between lidocaine and placebo overall. Thus, it can be concluded that missing follow-up data may not significantly affect the outcome.

It had been shown that neuroinflammation is correlated with the occurrence of POCD [34]. It is believed that the BBB is formed by brain endothelial cells that line the cerebral microvasculature. The BBB is a vital mechanism that protects the brain from changes in the composition of plasma and circulating compounds capable of disrupting neuronal function [35]. The immune response and surgical trauma may trigger cellular damage; these cells begin to release endogenous molecules, exacerbating the inflammatory response [36]. The immune response can trigger vascular endothelial cell damage and interrupt tight junction proteins. The BBB breaks down, allowing and facilitating the entry of peripheral immune cells into the brain, which triggers or exacerbates the activation of glial cells and neuroinflammation [37].

In addition to the neuroinflammation mechanism, danger-associated molecular patterns released following surgical trauma may be another factor resulting in POCD. Danger-associated molecular patterns interact with pattern recognition receptors that are present within the BBB endothelium and further activate proinflammation [38]. The anti-inflammatory and immune protective effects of lidocaine have been reported in the literature [39, 40], which reduces the permeability of cell membranes to Na^+^, avoiding membrane depolarization [41]. Thus, it can be thought that lidocaine can inhibit the release of inflammatory cytokines and vascular permeability. Additionally, lidocaine inhibits neutrophil adhesion, migration and accumulation [42], macrophage activity, and enzyme release [43]. In other words, it can be inferred that lidocaine attenuated the incidence and severity of POCD by stabilizing the BBB membrane through ion exchange and inhibiting the inflammatory response.

The study conducted by Ghafari et al. [44], which focused on the effect of lidocaine on cognitive deficits after coronary artery bypass graft surgery, was not included in this meta-analysis. It included 110 patients scheduled for CABG with CPB. This demonstrated that lidocaine could improve postoperative cognitive outcomes compared with procaine. Although we did not set the inclusion criteria for the surgical procedure, we aimed to evaluate the effect of intravenous lidocaine, while the intervention agent was used in a cardioplegia solution in this study. Another study by Zhu et al. [25] compared a mixture of lidocaine and ketamine with normal saline. The mixture was used as a bolus, and lidocaine was continued. Although ketamine is a short-acting reagent, the anesthesia time in the study was approximately 3 h (236.2 ± 41.7 min and 233.7 ± 38.2 min in lidocaine and placebo, respectively). Most importantly, a meta-analysis revealed that ketamine did not change the incidence of POCD [45]. Therefore, we included this study in the meta-analysis. However, caution should be exercised when interpreting the outcomes.

Although some have argued that the Jadad score is a simplistic measure that does not characterize all elements of trial quality, it is still perhaps the most common measure of trial quality for assessing the methodological quality of a trial [17].Blinding, randomization, and description of dropouts are the three basic minimum assessment tools before inclusion of trials in meta-analysis. It is known to have good validity and reliability. Its brevity and ease of use makes it one of the most widely used scales, and it offers the prospect of objectivity, which is much more efficient than some other subjective methods. The use of the modified Jadad score thus helped to avoid misinterpreting the quality of studies.

This meta-analysis had several limitations. First, some discrepancies are attributable to the use of different tests and the assessment of diverse populations. To balance the bias of the different tests, we used the difference in values compared with the baseline. Second, we performed a meta-analysis of the data from the study conducted by Peng et al.[24], which used several tests to assess POCD at different follow-up times. Although the study quality was high (Jadad score of 5), it may have deteriorated weight bias. Third, the surgical procedure was not an independent risk factor for POCD, and we included 4 (out of 10) trials that underwent cardiac surgery. Prolonged hospitalization and increased resource use may be associated with neurobehavioral declines [46, 47]. Finally, the total dose of lidocaine was not set the same as different regimens were used in studies. Different dose of lidocaine may aggravate the bias in different studies.

Taken together, multiple studies have demonstrated that POCD occurring in the short term is predictive of late cognitive decline [29, 48, 49]. In this systematic review and meta-analysis, we found that lidocaine could alleviate the overall incidence of POCD in the short and long term, especially the occurrence and severity in the short term. Thus, lidocaine can be a valuable preventive intervention to significantly reduce the risk of both short term and long term POCD. Most eligible studies did not find any significant differences on long term POCD. It may be because these studies were underpowered to detect an effect on long term POCD as there are more confounders. It warrants further studies on long term POCD.

### Electronic supplementary material

Below is the link to the electronic supplementary material.

Supplementary Material 1

## Data Availability

The datasets used and analysed during the current study available from the corresponding author on reasonable request.
