# Supplementary material for: The effect of intravenous lidocaine on postoperative cognitive dysfunction: a systematic review and meta-analysis
Source: BMC Anesthesiol. 2023 Sep 5;23:299. doi: 10.1186/s12871-023-02202-0 (PMC10478315; doi:10.1186/s12871-023-02202-0)
Supplement: Supplementary file 1 — Supplementary Material 1 [file 12871_2023_2202_MOESM1_ESM.doc]

# The appendix: A- The search strategy in MEDLINE databases: (1) Cognitive Therapy [MeSH] (2) Cognition Disorders [MeSH] (3) Cognition [MeSH] (4) Neuropsychology [MeSH] (5) Neuropsychological Tests [MeSH] (6) Cognitive Impairment [MeSH] (7) delirium [MeSH] (8) postoperative cognitive dysfunction [MeSH] (9) 1 OR 2 OR 3 OR 4 OR 5 OR 6 OR 7 OR 8

# (10) Lidocaine [MeSH]

# (11) Xylocaine [MeSH] (12) Lignocaine [MeSH]

# (13) 10 OR 11 OR 12 (14) 9 AND 13

# B- Quality of GRADE evidences and included articles:

GRADE provides a clearly articulated, comprehensive, and transparent methodology for rating and summarising the quality of evidence supporting management recommendations. [1]. Quality of evidence as used in GRADE system means more than risk of bias and so may also be including the overall risk of bias, inconsistency, indirectness, imprecision, and publication bias [2]. The following factors were used to deﬁne the quality of evidence: high quality, further research is unlikely to change the conﬁdence in the estimate of effect; moderate quality, further research is likely to have an important impact on the conﬁdence in the estimate of effect and might change the estimate; low quality, further research is likely to have an important impact on the conﬁdence in the estimate of effect and is likely to change the estimate; and very low quality, we are uncertain about the estimate [1-2]. Decisions on quality of evidence were made using guidance from GARDE publications and website [2-10]. The GRADE working group is available at ; https://www.gradepro.org/ (Accessed April 2023).
Procedures for Assess the quality of evidence based on GRADE synthesis:

A-Study design: Randomized Clinical Trials (RCTs), and non-RCTs. The concluding of evidence was based on RCTs if presented. If the RCTs were not available, the evidence would concluded by non-RCTs.
B- Study quality: GRADE scale for RCTs. We adapted the following GRADE Items; Item1: Random allocation, Item2: Concealed allocation, Item 4&5: Blinding of participants and therapists, Item 6: Blinding assessors, Item 7: Adequate follow-up, and Item 8: Intention to treat analysis.

C- Consistency: refers to the similarity in effect across the studies. It can be calculated statically by the I^2^ heterogeneity item according the meta-analysis.
D-Directness: refers to the extent to which people, intervention and outcome measures are similar to those of interest. Four types of directness were assessed: Directness in population, interventions, comparisons and outcome.
E- Publication bias: examined using egger regression test, with a significant publication bias considered to be *p*≤ .10 [6].

Type of evidence and GRADE:
Randomize clinical trials: High
Observational studies: Low

Decrease grade if
1-Serious (-1) or very serious (-2) limitation to study quality.
-For RCT in our present study: Serious limitation is problem with 1 element on the GRADE items and very serious limitation is problem with 2 elements or more on the GRADE items.
In case of Item 7 was (no) on GRADE scale; if the authors explained the reason of withdraw, no down grade in the quality regarding this item.

Increase grade if:
For RCT in our present study:
1-Large effect size (+1): significant relative risk with effect size >2 or <0.5 and very large effect size (+2): significant relative risk with effect size >5 or <0.2.
2- Evidence of a dose response gradient (+1).
3-All plausible confounders would have reduced the effect (+1).

References
1. Elena R. GRADE: what is “quality of evidence” and why is it important to clinicians?. Bmj. 2008. 3;336:995
2. Guyatt G, Oxman AD, Akl EA et al. GRADE guidelines: 1. Introduction—GRADE evidence profiles and summary of findings tables. J clin epidemiol. 2011; 30:383-94.
3. Guyatt GH, Oxman AD, Kunz R et al. GRADE guidelines: 2. Framing the question and deciding on important outcomes. J clin epidemiol. 2011; 64: 395-400.
4. Balshem H, Helfand M, Schünemann HJ et al. GRADE guidelines: 3. Rating the quality of evidence. J clin epidemiol. 2011; 64:401-6.
5. Guyatt GH, Oxman AD, Vist G et al. GRADE guidelines: 4. Rating the quality of evidence—study limitations (risk of bias). J clin epidemiol. 2011; 64:407-15.
6. Guyatt GH, Oxman AD, Montori V et al. GRADE guidelines: 5. Rating the quality of evidence—publication bias. J clin epidemiol. 2011; 64:1277-82.

7. Guyatt GH, Oxman AD, Kunz R et al. GRADE guidelines 6. Rating the quality of evidence—imprecision. J clin epidemiol. 2011; 64:1283-93.

8. Guyatt GH, Oxman AD, Kunz R et al. GRADE guidelines: 7. Rating the quality of evidence—inconsistency. J clin epidemiol. 2011; 64:1294-302.

9. Guyatt GH, Oxman AD, Kunz R et al. GRADE guidelines: 8. Rating the quality of evidence—indirectness. J Clin Epidemiol. 2011;64(12):1303-10.
10. Guyatt GH, Oxman AD, Sultan S, et al. GRADE guidelines: 9. Rating up the quality of evidence. J clin epidemiol. 2011; 64:1311-6.
